# Sympathetic science: analogism in Brazilian ethnobiological repertoires among *quilombolas* of the Atlantic forest and Amazonian *ribeirinhos*

**DOI:** 10.1186/s13002-021-00499-0

**Published:** 2022-01-03

**Authors:** Helbert Medeiros Prado, Rui Sérgio Sereni Murrieta, Glenn Harvey Shepard, Tamires de Lima Souza, Marcelo Nivert Schlindwein

**Affiliations:** 1 Philosophy and Social Sciences Institute, Federal University of Pará, 01 Augusto Corrêa Str., Belém, PA 66075-110 Brazil; 2 Bioscience Institute, University of São Paulo, 277 Matão Str., São Paulo, SP 05508-090 Brazil; 3 Department of Anthropology, Emilio Goeldi Museum, 1901 Perimetral Av., Belém, PA 66077830 Brazil; 4 Center of Sciences and Technology for Sustainability, Federal University of São Carlos, João Leme Dos Santos, Highway 110km, Sorocaba, SP 18052-780 Brazil; 5 Department of Ecology and Evolutionary Biology, Federal University of São Carlos, Washington Luís Highway 235km, São Carlos, SP 13565-905 Brazil

**Keywords:** Ethnoecology, Environmental anthropology, Phenomenology, Epistemology, Ontology

## Abstract

**Background:**

Drawing on Phillipe Descola’s comparative analysis of ontological regimes across cultures, this article identifies *analogism* guiding ethnobiological repertories among two distinctive traditional tropical forest communities in Brazil.

**Methods:**

We carried out participant observation, semi-structured interviews and informal dialog with 48 individuals, among *quilombolas* of the Atlantic Forest in southeastern Brazil and *ribeirinhos* of the Amazon.

**Results:**

We documented 60 traditional practices governed by analogical principles, comprising hunting, ethnomedical practices, food taboos, and other interactions with non-human entities. We also identify and classify the analogical principles reported in the field data. Based on this classification, we address the phenomenological dimension of the ethnobiological repertoires and discuss the epistemological and ontological foundations of this form of reasoning. We also hypothesize on the role of analogism shaping ethnobiological repertories more generally in Brazil.

**Conclusion:**

The heuristic model we apply—articulating phenomenology, epistemology and ontology—could prove valuable in ethnobiology and the emerging field of “anthropology beyond the human.”

## Background

### Introduction

This article discusses the analogical reasoning shaping uses and practices involving fauna and flora among traditional *quilombola* communities of the Atlantic Forest and *ribeirinhos* of the Brazilian Amazon. The ethnobiological repertoires we analyze comprise food taboos, pregnancy and post-partum restrictions, medical practices, interventions aimed at improving humans’ and dogs hunting skills, and other interactions with non-human entities. These practices are referred to by local people at both fieldwork sites as *simpatias* (“sympathies”), and outsider-anthropologists might describe them as “sympathetic magic.” We avoid the term “magic” in our discussion, however, paying heed to Arthur C. Clarke’s [1] admonition that magic is just another word for technology.

In this article, we analyze analogism in these ethnobiological repertoires taking into account phenomenological, epistemological, ontological and historical components. The phenomenological dimension of our analysis consists in accessing sensory inputs impacting individuals’ ethnobiological repertories about nature and its uses [2–4]. We approach epistemology as an analytical tool to reveal the elemental structures of reasoning and cultural models shaping knowledge systems [5, 6]. Moving from epistemology to ontology, we apply Descola’s ontological framework to the analogical reasoning of the ethnobiological repertoires documented [7]. By ontology, we mean deeply rooted, non-discursive cultural assumptions about reality and the nature of relationships between humans and other beings of the cosmos, as persons, e.g., having agency and personality as do a person [8, 9]. In ethnobiology, analyzing the ontological dimension of local repertories represents an opportunity to better understand cultural knowledge regimes [10, 11]. Dialogue with the so called “ontological turn” in anthropology is still incipient in ethnobiology [4, 7, 12–20]. Finally, we develop a preliminary historical hypothesis to explain certain similarities in the analogical reasoning systems inherent in these two geographically distant and culturally distinctive Brazilian societies.

### Theoretical approach

As a philosophical tradition, phenomenology breaks from the Cartesian intellectualist paradigm and reintroduces the experience of the concrete world as a starting point for understanding human knowledge. To phenomenologists, perception is conceived as an immediate, sensory, and non-reflective instance of human experience, which precedes and sustains any rational (or intellectual) expression of knowledge [21, 22]. Epistemology, in its turn, can be described as a systematic philosophical and scientific investigation about knowledge systems, in terms of their premises, concepts, and rationalities. By ontology, we mean a deeply rooted philosophical subject, which has to do with inquiries involving the nature (or essence) of beings and things, and their relationship to humans [8]. Below, we will introduce the way we mobilize these concepts into our ethnobiological case study.

Following Ingold [15], we focus on the phenomenological dimension of local knowledge that emerges from the empirical engagement of individuals with species of flora and fauna, amongst other environmental features [23]. Phenomenology is also relevant to forms of knowing immersed in a particular sociocultural context of knowledge acquisition or “prehension”, as conceptualized by Roy Ellen [24]. By “prehension” Ellen means the human classification of nature as a situational (and contingent) process, “based on the life experiences” [10, p. 93] of individuals, rather than the expression of an abstract or supposed universal taxonomic model. More specifically, in this article, we will discuss the phenomenological dimension of our results in the light of “ecological apparency” [25, 26] and “sensory apparency” [2] hypotheses, and to which we add our own novel concept of “ethological apparency.”

We approach epistemology in our analysis proposing a critique and an update to the predominance of folk taxonomy in classic ethnobiological studies [24, 27–33], focusing instead on the principle of resemblance as a criterion for inferences about affinities between organisms [33–36] and cultural use categories [37–39]. The ontological perspective in anthropology, in its turn, has criticized the universality of the Western nature-culture dichotomy, especially in relation to non-Western or indigenous systems of thinking [15, 40–42].

Among the prominent authors associated with the “ontological turn” in anthropology, Philippe Descola [7, p. 121] proposes a classificatory and typological schema to address and recognize different ontological regimes in the present and past societies. Descola’s model is focused on cultural understandings of continuity (identity) vs. discontinuity (alterity) between humans and non-humans, crossing this dichotomy with the binary distinction between exteriority (physicality or material appearance) and interiority (the subjective essence, agency, or intentionality) of things and beings (entities) to produce a four-fold classification of ontological regimes: naturalism, animism, totemism, and analogism.

In naturalism, which according to Descola is restricted to modern Western thinking, all entities in the world are unified by physicality (i.e., the same underlying materiality) but distinguished by interiority, with only humans expressing subjective intentionality. In animism, all entities are linked by the same interiority (all beings have subjective intentionality), but are dissimilar in terms of exteriority (i.e., the physical bodies of living beings). This ontological regime has been described by Eduardo Viveiros de Castro [14] as “perspectival multinaturalism” and is associated with lowland South American indigenous philosophies. In the case of totemism, found for example among Australian Aborigines [43–45], all entities in a given collective have the same origin, being composed both externally and internally of the same underlying essence [7]. Finally, in analogism, both the physicality and interiority of each entity in the world are distinct and therefore treated as unique [7, p. 121].

According to Descola [7], in an analogical ontology, even though each entity in the world is unique in its exteriority (materiality) and interiority (subjectivity), their qualities are exchangeable. To Descola, precisely because the world is so segmented (in terms of each entities’ uniqueness) people are compelled to mobilize (and connect) different entities in a logical system as a way of making the world intelligible [7, p. 202]. It is this aspect of analogism in particular that we draw on as on ontological model for the sympathetic practices reported in our study areas. As discussed below, the external apparencies of plants, animals, and other objects, or their parts, are manipulated by people toward practical ends. Our study builds on classical anthropological analyses of sympathetic magic [44, 46–56], while bringing these perspectives into dialogue with contemporary approaches to human–nature interactions such as “multispecies ethnography” [57], “multispecies landscapes” [58, 59], “anthropology beyond human” [42], and “anthropology of life” [60, 61] as well.

In addressing analogism as observed among the study communities, we do not exclude the possibility of other ontological regimes in operation in the two ethnographic contexts. As proposed by Descola, the four ontological models are not mutually exclusive, and are sometimes found side by side in the same society. Moreover, Sahlins [62] has argued that analogism can also be conceived of under the umbrella of a broader conception of animism. We focus on Descola’s analysis of analogism, however, since it seems to describe so well the phenomena we observed in the field, and because it has been so rarely addressed in a systematic way in ethnobiological repertoires. While some authors have argued against strictly materialistic analyses of medicinal plant usage, noting the widespread occurrence of the “Doctrine of Signatures” [26, 47, 63] our study is the first we are aware of to probe deeper into the ontological basis of these practices using Descola’s model.

In his comparative analysis, Philippe Descola identifies analogism among societies in China, Africa, and pre-Colombian Mesoamerica, as well as in medieval and Renaissance Europe. This geographical association enters into our historical analysis as we raise the following question: could analogism have become pervasive in non-indigenous ethnobiological repertories in Brazil via Portuguese colonization? We evaluate this question in the Discussion, hoping to stimulate new research into the epistemic regularities observed in these Brazilian ethnobiological repertories, and their possible historical and geographical origins. Finally, we outline how ethnobiologists might use this analytical framework to better evaluate the multiple layers of knowledge and practice found in ethnobiological repertories, including phenomenological, epistemological, ontological and historical dimensions.

## Methods

### The quilombola context (Atlantic Forest, southeast Brazil)

**Fig. 1:**
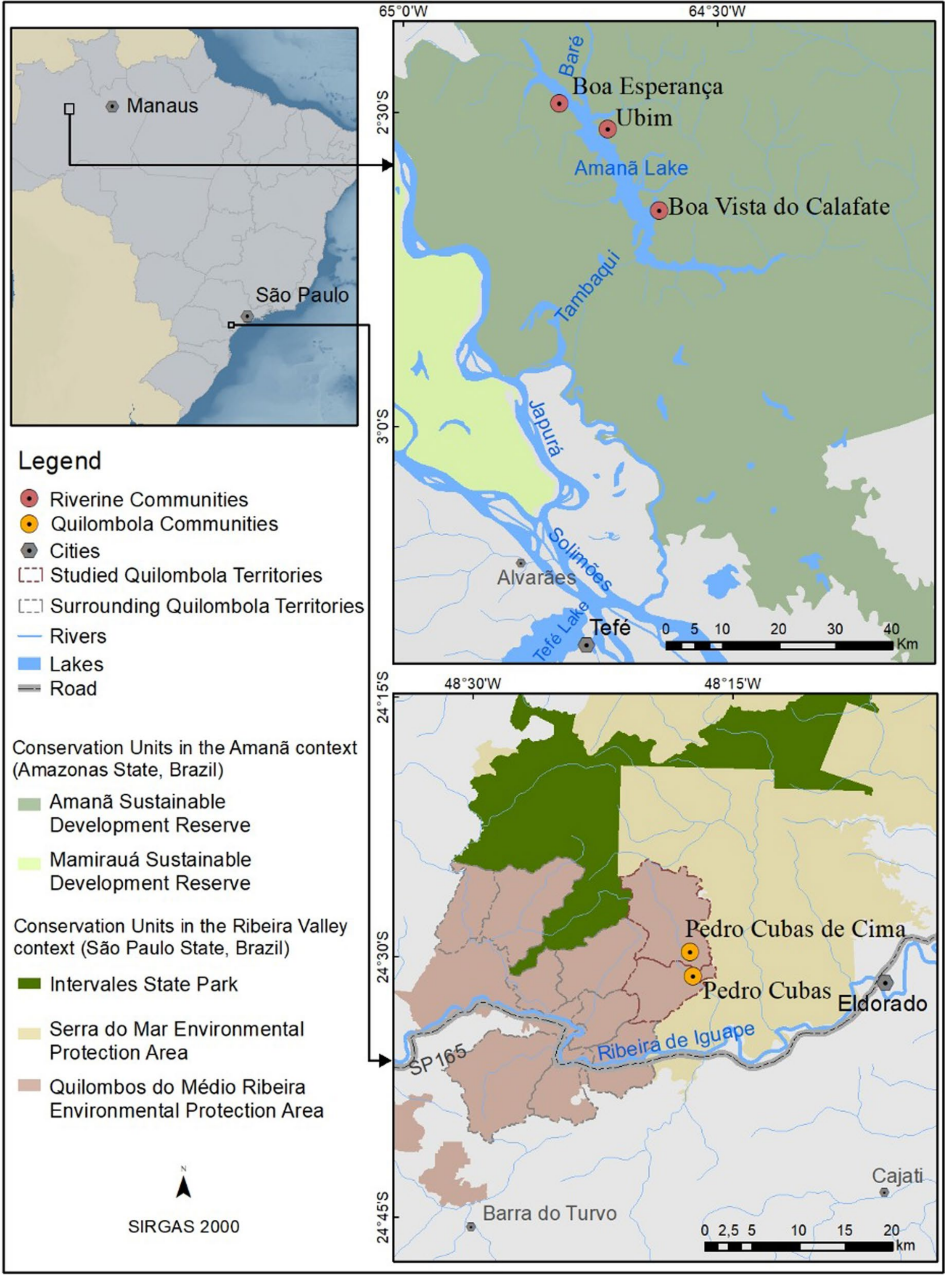
Study communities and surrounding areas in the Amanã Reserve (above) and Ribeira Valley (below)

The Ribeira valley is located in southeast Brazil, between the states of São Paulo and Paraná, occupying an area of 2,830,666 ha [64] (Fig. 1). The climate of the region is tropical (Af), and approaching a humid subtropical (Cfa) climate, based on the Köppen classification system adapted for Brazil [65]. The Ribeira valley is UNESCO natural World Heritage site and forms part of the largest continuous area of Atlantic Forest (dense montane/submontane rainforest) in Brazil [66, 67].

The *quilombola* communities of the middle Ribeira valley were founded by escaped, freed or abandoned slaves during the colonial period in Brazil, from the late eighteenth century to the early nineteenth century [68]. Slash-and-burn cultivation (*coivara*) supplemented by animal husbandry has been the main mode of food production of these populations since their origins, also representing an important element of cultural identity among them [69–72].

Over the past half century, important socio-cultural changes have marked the quilombola population in the region. Disputes over land, economic pressure, and the imposition of environmental legislation, in addition to overlap with protected areas, have discouraged some traditional practices, especially “slash and burn” swidden cultivation [69]. Greater involvement with NGOs, universities and government agencies have also brought about economic and social transformations [70, 71, 73]. In the religious sphere, traditional syncretism between folk Catholicism combined and African spirituality [74, 75] has been increasingly impacted by the presence of Evangelical Christian denominations within quilombola territories [75].

In terms of domestic economy, the population is currently composed mostly of family farmers who also practice small-scale extractivism and subsistence hunting [76]. Other sources of cash income include government programs for poverty relief and rural pensions [64, 70]. Data presented here were gathered during ethnographic field studies conducted in the communities of Pedro Cubas and Pedro Cubas de Cima (Fig. 1). The territory of Pedro Cubas covers 3,806 ha, with a population of approximately 150 people in 40 household units [36; 2005 data]. Pedro Cubas de Cima has a recognized area of 6,875 ha. The population comprises approximately 120 people distributed in approximately 30 households [36; 2005 data].

### The ribeirinho context (Central Amazonia)

Created in 1998 and located between the Negro and Japurá river basins, the Amanã Sustainable Development Reserve (ASDR), totaling 2,350,000 ha, is one of the largest protected tropical forest areas in South America. Upland *terra firme* forest predominates, with smaller areas of white-sand savanna (*campinarana*) and seasonally flooded *várzea* (white water) and *igapó* (black water) forests [77]. The climate is tropical (type Af in the Köppen classification), with high mean monthly temperatures and high rainfall throughout the year [65].

Amanã lake is 45 km long and 2–3 km wide (Fig. 1) and harbors most of the reserve’s inhabitants on its shores and tributaries. The region is subject to seasonal water pulses that lead to river level variations of 9–10 m between the rainy (November to July) and dry (July to November) seasons. The flooding regime has a direct influence on local populations seasonal strategies of landscape use and resource capture, modulating their domestic economy throughout the year [78].

Currently, ASDR is home to approximately 5,000 inhabitants, distributed across 124 communities (or villages) and independent (or isolated) households [47, 2018 demographic census]. The population includes families of Amazonian origin, typically known as *caboclos*, as well as migrants from northeast Brazil, referred to in the region as *arigós* [79]. The formation of so-called *caboclo* society reaches back to the early Portuguese colonization of the Amazon, including a strong indigenous component in its constitution [80–83]. The *arigós*, on the other hand, came to the region as part of migrations from northeast of Brazil during the Rubber Boom in Amazonia [84]. The first generation of *arigós* in Amanã would have initially migrated to the upper Juruá (Fig. 1) at the turn of the twentieth century, coming only later to Amanã lake in search of unexploited lands [79].

Several authors have pointed out the analytical limitations and racism inherent in the concept of *caboclo* [85, 86], so we use the more neutral term *ribeirinho* (“river-dweller”) to refer to the population of ASDR. Historically, folk Catholicism in combination with indigenous cosmologies form the main religious matrix in Amanã. Catholic priests of the “liberation theology” movement were important figures in the political organization of these communities beginning in the 1960s [87]. In a recent survey, about 45% of the population of Amanã declared themselves Catholic, 35% as Evangelical, while the remaining 20% did not declare religious affiliation [50; data from 2018].

Today, the *ribeirinhos* residing in ASDR subsist on a mixed economy based on agriculture, fishing, hunting and extractivism, and may benefit from federal poverty relief programs [88]. Research for this study was conducted in three communities located on the shores of lake Amanã: Boa Esperança, Ubim and Boa Vista do Calafate (Fig. 1). Boa Esperança, the largest community in Amanã, is composed of 300 residents in 71 households. Boa Vista do Calafate, with 90 residents, is composed of 15 households, while Ubim comprises 35 residents in 7 households [50; data from 2018.]

### Fieldwork and ethnographic approach

The analogies reported in this study were recorded during semi-structured interviews and informal conversations during ethnographic research on hunting practices, pregnancy and post-partum food taboos, and other aspects of ethnoecological knowledge. The semi-structured interview protocol included the following guiding topics [89]: (1) life history; (2) gender roles, domestic economy, natural resource harvesting and usage; (3) landscape perception; and (4) food restrictions and taboos. Informal conversations, facilitated by ethnographic immersion and participant-observation in daily activities, were systematically recorded in field diaries.

Local terms appear in the text and tables in italics, accompanied by an English gloss where one exists. Taxonomic nomenclature (species, genus or family) is mentioned only upon first appearance. We identified plants using botanical identification guides of native and exotic species in Brazil [90–92]. We identified vertebrate animals using taxonomic guides of the Brazilian fauna [93–96], including literature specific to southeastern Brazil and the Ribeira valley [97–99], as well as to the Amazonian fauna of ASDR [78, 100]. We made no attempt at accurate taxonomic identification of invertebrate species.

In the Ribeira, observations were recorded during five expeditions for a total of 45 fieldwork days between December, 2018 and January, 2020, comprising 24 participants: sixteen women and eight men between 35 and 82 years of age. Observations at Amanã resulted from two visits and a total of 30 fieldwork days between January and March, 2019, with 24 participants: fifteen women and nine men between 24 and 79 years of age. Despite the difference in fieldwork effort, we made sure to include a similar number of respondents and a similar gender ratio between the two study regions. Observations were recorded by the same interviewers in both study areas (HMP and TLS).

Participants were selected based on an adaptation of the “snowball” technique [101], whereby experienced residents, appraised of the research objectives, helped identify other residents with the desired knowledge profile for the study. Considering the potential for interference due to gender differences between researchers and respondents, fieldwork was organized so that male author HMP worked with men and female coauthor TLS worked with women. In this article, pseudonyms preserve the participants’ anonymity.

## Results

### Preliminary ethnographic findings: an emergent hypothesis

“The huntress wasp, she catches spiders and takes them to her burrow... So you kill the wasp and roast it and give it to the dog to eat... Then the dog will be good at hunting burrowing animals... armadillo, peccary, things that hide in holes like that.” (Zeca, December 7, 2018; authors’ translation).

In the initial days of fieldwork in the Ribeira valley (São Paulo, Brazil), author HMP was talking with Zeca (male, 58 years old) about using dogs to locate and capture game animals. At one point, Zeca mentioned a tried and true remedy for making dogs into fierce hunters: feeding the dog a powder made by roasting and grinding a certain wasp species, which he referred to as *caçadeira* (‘huntress’). When asked what was special about this insect for it be used in this way, Zeca provided the explanation cited above. For Zeca, feeding the dog powder made from the ‘huntress’ wasp makes it better able to hunt burrowing animals, such as *tatu* (armadillo; Dasypodidae), *cutia* (agouti; *Dasyprocta azarae*) and *paca* (*Cuniculus paca*).

Later, Zeca described another treatment for hunting dogs that involved taking a leaf of the *embaúba* tree (*Cecropia sp*.), drying it over a fire or in the sun and then burning it and blowing the smoke over the dog. This treatment improves the dog’s ability to corner animals that, like the *Cecropia* leaf, “live up high,” namely arboreal animals such as *quati* (coatimundi; *Nasua nasua*), primates, and highly sought-after bird species such as *jacu* (guan; *Penelope sp*.) and *jacutinga* (black-fronted piping guan; *Aburria jacutinga*).

At this point, researcher HMP became interested in the logical principles structuring these and other uses of animals and plants among this population. The same relational principle appeared to underlie these two use reports: the burrow of the *caçadeira* wasp was likened to the burrowing habit of certain game animals, while the height from which the *Cecropia* leaf falls was likened to the arboreal habits of certain other game species.

These and other uses and practices revealed different inhabitants’ intellectual effort to identify behavioral or ecological similarities between organisms as biologically distinctive as wasps and dogs, or trees and arboreal animals. Such correspondences, in turn, provide a mechanism for the transmission and acquisition of certain desirable qualities between entities that we refer to as *emanators* and *receivers*. Both treatments described by Zeca reveal relationships forged perceptually and intellectually by a general principle of “sympathetic” or analogical reasoning.

### Analogies documented

Based on this revelatory incident, we began to explore the principles guiding a wide range of plant and animal uses, adapting our initial ethnographic study (focused on livelihood strategies) to include systematic reports of such analogical practices in the two study regions. In the ensuing work, we recorded 60 different examples of analogical rationality related to different plant and animal uses and practices observed in current daily activities (Table 1).

**Table 1:** Analogies reported in the study regions of Amanã (ASDR) and Ribeira valley (RV)

No.	Topic/site	Short description of analogies
1	Hunting/ASDR	Administering predatory beetle^1^ to a dog improves hunting ability
2	Hunting/ASDR	Administering animals with a keen sense of smell to a dog improves olfactory capacity
3	Hunting/ASDR	Administering the ear and snout of a *cutia* (agouti)^2^ to a dog improves the dog’s hearing and sense of smell in pursuit of prey
4	Hunting/ASDR	Administering brain (*miolo*) of the *japiim* (yellow-rumped cacique)^3^, considered intelligent due elaborate nests, makes the dog more intelligent
5	Hunting/ASDR	Administering the brain of primates, considered intelligent, makes the dog more intelligent
6	Hunting/ASDR	Administering the brain of *calango* (lizard)^1^, considered a good predator, makes the dog a better hunter
7	Hunting/ASDR	Administering brain of *urubu* (vulture)^4^, which “sees prey from afar”, improves dog’s hunting ability
8	Hunting/ASDR	Administering the tooth of *boto* (Amazon river dolphin)^5^ or *tucuxi*^6^, “which hunt a lot”, improves a dog’s hunting ability
9	Hunting/ASDR	Administering ants^1^ that “walk in groups” improves the dog’s ability to chase herds of *caititu* (peccary)^7^
10	Hunting/ASDR	Administering *morcegos* (bats)^1^ that “roost in a row” improves the ability of the dog to corner herds of *caititu* (peccary)^7^
11	Hunting/ASDR	Administering *caba* (wasp)^1^, because it is a predator (a hunter), makes dog a better hunter
12	Hunting/ASDR	Depositing a small piece cut from a dog’s ear on prey tracks improves the ability of the dog to chase prey
13	Hunting/ASDR	Depositing a dog’s fur on prey tracks improves the dog’s ability to chase prey
14	Hunting/ASDR	Depositing a dog's fur on *onça* (jaguar or puma)^8^ tracks improves the ability of the dog to chase jaguars or pumas
15	Hunting/ASDR	Blowing the smoke of burnt *onça* (jaguar or puma)^8^ fur on a dog improves the dog’s ability to chase jaguars or pumas
16	Hunting/RV	Administering a powder made from burnt *caçadeira* (huntress wasp)^1^ that is predatory and burrows, improves the dog’s ability to hunt burrowing animals
17	Hunting/RV	In addition to practice 16, the powder should be placed in a single point in the dog’s food so that the dog can go directly to where the prey is. If the powder is spread, the dog will lose its prey
18	Hunting/RV	Administering feathers of *urubu* (vulture)^4^, considered an excellent detector of prey, improves the ability of a dog to detect prey from afar
19	Hunting/RV	In addition to practice 18, the *urubu* (vulture)^4^, by feeding on dead animals, causes the dog to die early
20	Hunting/RV	When the smoke from burnt *embaúba* (*Cecropia*)^9^ leaf (located up high) is blown on a dog, the dog is better able to corner arboreal animals
21	Hunting/RV	When butchering *bugio* (howler monkey)^10^, some hunters keep the animal’s sac-shaped hyoid bone, responsible for vocalization. Depositing chili pepper in the stored hyoid bone irritates the throat of howler monkeys in the forest, making them easier to hunt
22	Hunting/RV	The hunter holds a tree leaf next to the shotgun when shooting a *bugio* (howler monkey)^10^: when shot, howler monkeys attempt to heal themselves by rubbing leaves on their wounds
23	Childbearing/ASDR	Burying the umbilical cord near the mother’s home accelerates the time to her next pregnancy (according to some reports, the opposite is also true)
24	Childbearing/ASDR	It is locally recognized that the *umbigo da castanha* (operculum of the Brazil nut)^11^ does not pass through the orifice of the pericarp and, thus, can only be accessed by cutting the pericarp open (Fig. 3). Throwing a Brazil nut operculum behind a pregnant woman's back causes the child to “stay stuck in her belly”, preventing natural childbirth
25	Childbearing/ASDR	If a pregnant woman eats a *jabuti* (red-footed tortoise)^12^, which is a slow animal, labor will be prolonged
26	Childbearing/ASDR	If a pregnant woman eats *pato* (duck)^13^, which frequently defecates and has “soft stool”, the baby will have severe diarrhea
27	Childbearing/ASDR	If a pregnant woman eats *jabuti* (red-footed tortoise)^12^, which retracts its head, the fetus will also retract in the mother's uterus, making natural childbirth impossible
28	Childbearing/ASDR	If a pregnant woman eats meat from an animal that was difficult to kill, it will lead to difficult childbirth (according to some reports the opposite is also true)
29	Childbearing/ASDR	An injury or disfigurement caused to an animal during a hunt by the father will manifest in the newborn child
30	Childbearing/ASDR	If a hunter unnecessary injures an animal, his child is born with the appearance of the mistreated animal
31	Childbearing/ASDR	The same suffering experienced by an animal mistreated by a pregnant woman will manifest in the child
32	Childbearing/ASDR	A harming caused by a pregnant woman to a dead animal (carcasses) will manifest in the newborn
33	Childbearing/ASDR	If a father exerts physical effort in any activity during his wife’s pregnancy, the child will also exert a lot of physical effort and suffer as a consequence
34	Childbearing/ASDR	The bark of trees that regenerates quickly is applied to the genitalia of women after childbirth to speed recovery
35	Childbearing/RV	If a pregnant woman eats an animal with large claws, the child will “scratch her belly”
36	Childbearing/RV	Eating *animal de casco* (turtles), which retracts its head, causes the child to retract during childbirth
37	Childbearing/RV	If a pregnant woman eats *tatu* (armadillo)^14^ tail, considered large by the locals, the child will be born with a large penis
38	Childbearing/RV	During *resguardo* (immediate postpartum period) a woman cannot eat prey killed in a *mundéu* (a trap that crushes the animal with tree trunks) "because the child’s guts will come out, like what happens with an animal in a *mundéu*"
39	Childbearing/RV	Eating an animal killed in a *mundéu*—a trap that crushes the animal – will cause back pain in women who have recently given birth
40	Childbearing/RV	If a lactating woman eat female *veado* (deer)^15^ meat “her milk will dry” (she won’t have more milk)
41	Childbearing/RV	A woman who has recently given birth who eats *jacutinga* (black-fronted piping guan)^16^, which has white feathers on its head, will get gray hair early
42	Childbearing/RV	Eating a domestic pig^17^ that has already given birth and that has a lump (or inflammation) in the uterus causes the same problem in women who have recently given birth
43	Childbearing/RV	Eating *galinha botadeira* (laying hen)^18^ with a lump (or inflammation) in the uterus causes the same problem in women who have given birth
44	Childbearing/RV	Eating fish^1^ with reddish eyes gives women who have recently given birth reddish eyes
45	Childbearing/RV	Eating bloody meat causes hemorrhaging in women who have recently give birth
46	Childbearing/RV	Eating an adult rooster^18^ can make its song “get stuck in the head of a woman who has recently given birth, which can make her crazy”
47	Child health/RV	If a newborn wears yellow clothes, they will get *amarelão* (“jaundice” associated locally with hookworm infection)
48	Child health/RV	Twisting a newborn's diaper causes the baby to have stomach pain and diarrhea
49	Virility/ASDR	The broth from *jabuti* (tortoise)^12^, which has a retractable head, retracts the penis of the man who drinks it
50	Virility/RV	A man who eats a preparation made with the reproductive organ of coati^20^ (grated and mixed with rum) will have better sexual performance
51	Virility/RV	Regarding practice 50, grating the organ from the bottom up leads to an erection, bottom down leads to impotence
52	Meteorology/ASDR	If an *ave coã* (laughing falcon)^21^ lands on a dry tree, it signals dry weather. If it lands on a leafy tree, it signals rain
53	Meteorology/RV	The weather conditions in the first 12 days of the year indicate how they will be throughout the entire 12 months of that year
54	Meteorology/RV	The moon *derramando* (“pouring”), that is, in a vertical or inclined position, indicates rain or cloudy weather
55	Other Medicines/ASDR	The use of *cachorro pelado* (pencil cactus)^19^ extract, due to the highly branched (articulated) architecture of its branches (Fig. 3), cures dislocated limbs
56	Other Medicines/ASDR	Eating watermelon^22^ (red and liquid) can cause bleeding in menstruating women
57	Other Medicines/RV	A person with a flesh wound who eats *tatu* (armadillo)^14^ will feel the wound scratching (because of armadillos’ long claws)
58	Other/ASDR	The relative physical weakening of a farmer weakens plants^1^ that he or she cultivates
59	Other/RV	Attaching the tip of the tail of a newly acquired dog to the post of the house prevents it from returning to its former home. In this kind of sympathetic belief, in which the part represents the whole, fixing part of dog in its new home is a way of not letting it run away
60	Other/RV	If a person destroys the tubular clay nest of a certain *vespa* (wasp)^1^, “everything they pick up is damaged”

1: Generic local term which may represent different scientific taxa; 2: *Dasyprocta fuliginosa*; 3: *Cacicus cela*; 4: Cathartidae; 5: *Inia geoffrensis*; 6: *Sotalia fluviatilis*; 7: *Dicotyles tajacu*; 8: *Panthera onca* or *Puma concolor*; 9: *Cecropia sp*.; 10: *Alouatta guariba*; 11: *Bertholletia excelsa*; 12: *Chelonoidis sp*.; 13: *Cairina moschata*; 14: Dasypodidae; 15: *Mazama sp*.; 16: *Aburria jacutinga*; 17: *Sus scrofa domesticus*; 18: *Gallus gallus domesticus*; 19: *Euphorbia tirucalli*; 20: *Nasua nasua*; 21: *Herpetotheres cachinnans*; 22: *Citrullus lanatus*

**Fig. 2:**
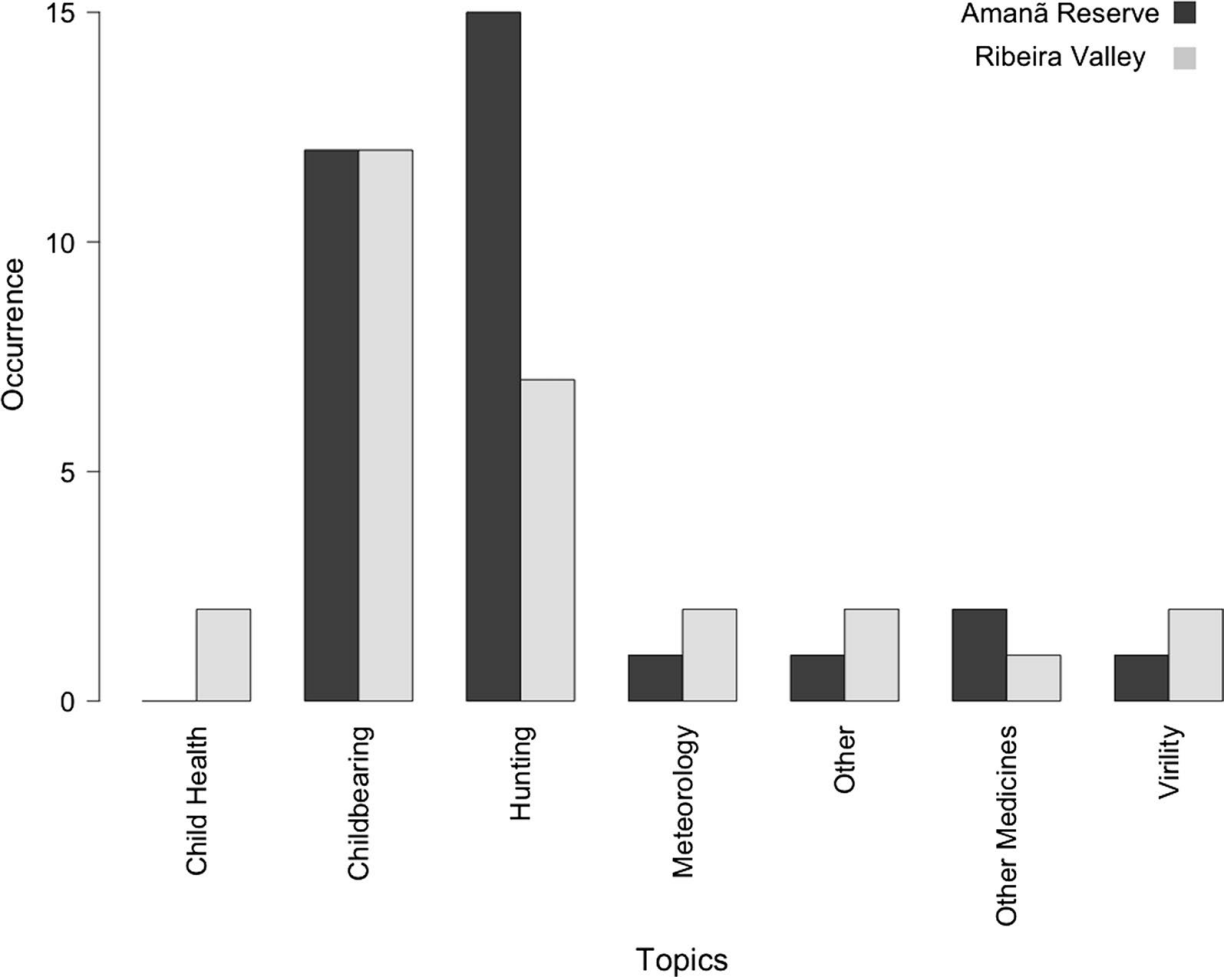
Occurrence of analogies by category and study region

Among the diversity of treatments, restrictions and other practices encountered, those involving pregnancy, childbirth and the post-partum period (grouped together under the category of “Childbearing”) accounted for 24 (40%) of the 60 analogical uses recorded (Fig. 2). Hunting applications accounted for another 22 (37%). Of these, most (20 of 22) were associated with honing the skills of hunting dogs. The prevalence of these topics results, in part, from our focus during fieldwork on men’s and women’s activities in both study sites. However these results also provide an indication of the importance and complementarity of these two central, gender-defined social roles [38].

### Sympathetic science

We identified five key components for analyzing the analogical models recorded: *correspondence* between parts, *activating feature*, *transmission* mode, *emanator* entity and *receiver* entity (Table 2). We developed this analysis based on our understanding of the essential explanatory logic underlying the uses and practices as reported by our interlocutors. While recognizing the overlap and juxtaposition with concepts proposed by various authors spanning more than a century of anthropological studies of “sympathetic magic” and other forms of non-Western rationality [44, 46, 49–56], we propose this conceptual model as an original contribution to inspire further studies. Specifically, we encourage ethnobiologists to carry out the same kind of systematic registration of sympathetic (or analogical) practices and rationalities as used for other aspects of ethnobiological knowledge.

**Table 2:** The five components of analogical interactions

Category	Description
*Correspondence*	Similarities among different entities defining the “sympathetic” relationship in analogical reasoning
*Activating feature*	A quality (*i.e*. morphology, behavior, etc.) that prompts an analogy
*Transmission*	The act that passes qualities between the analogous entities, ensuring a causal relationship
*Emanator*	Entity that transmits a quality
*Receiver*	Entity receiving the emanator’s quality

The five components defined above are linked as follows: sympathy or *correspondence* is identified or projected through a perceptual attribute (*activating feature*) that unifies two otherwise entities, and on this basis, an intervention is performed that establishes a causal relationship (*transmission*) between the parts, through which a quality is transmitted from one entity (*emanator*) to another (*receiver*).

We further identify ten categories of *activating* features: animal behavior (27 analogies; 45% of the total), physical state (7; 11.7%), morphology (7; 11.7%), part-whole relationships (6; 10%), function (6; 10%), preparation mode (3; 5%), and color, development stage, shape and lifestyle that were observed in one case each. The latter refers to specific requirements in preparing or administering a treatment to achieve the desired outcome, for example the importance of placing huntress wasp powder in a single spot in the dog’s food, rather than spreading it (Table 1, No. 17), or the direction of scraping affecting the outcome of male potency treatments (No. 51).

Transmission modes can be further distinguished according to whether they act through direct or indirect contact. Those that involve direct contact (ingestion, physical contact and inhalation) correspond to 75% of the recorded analogies. There are two cases that do not seem to involve a process of transmission of properties between the entities but, rather, more of an iconic signaling. These include the poetic metaphor of the “pouring moon” (No. 54), which signals rain, and the behavior of the laughing falcon (No. 52), which can indicate rain or drought depending on whether it lands on a leafy or dry tree.

A wide variety of emanator entities, especially animals, were recorded. These were present in 47 analogies (78.3% of the total) and include terrestrial and aquatic mammals, bats, reptiles, birds, fish, insects and domestic animals (*e.g.*, duck and chicken). Ten analogies (16.6%) contained plants or humans as emanators of properties, and three analogies were categorized as “Other” (5%) such as the “pouring moon” (No. 54) and clothing color (No. 47). In 33 analogies (55%), humans are the direct receivers, acting as indirect receivers in the remaining 27 analogies (45%).

## Discussion

### Phenomenological dimension: perceptual basis of environmental analogies

**Fig. 3:**
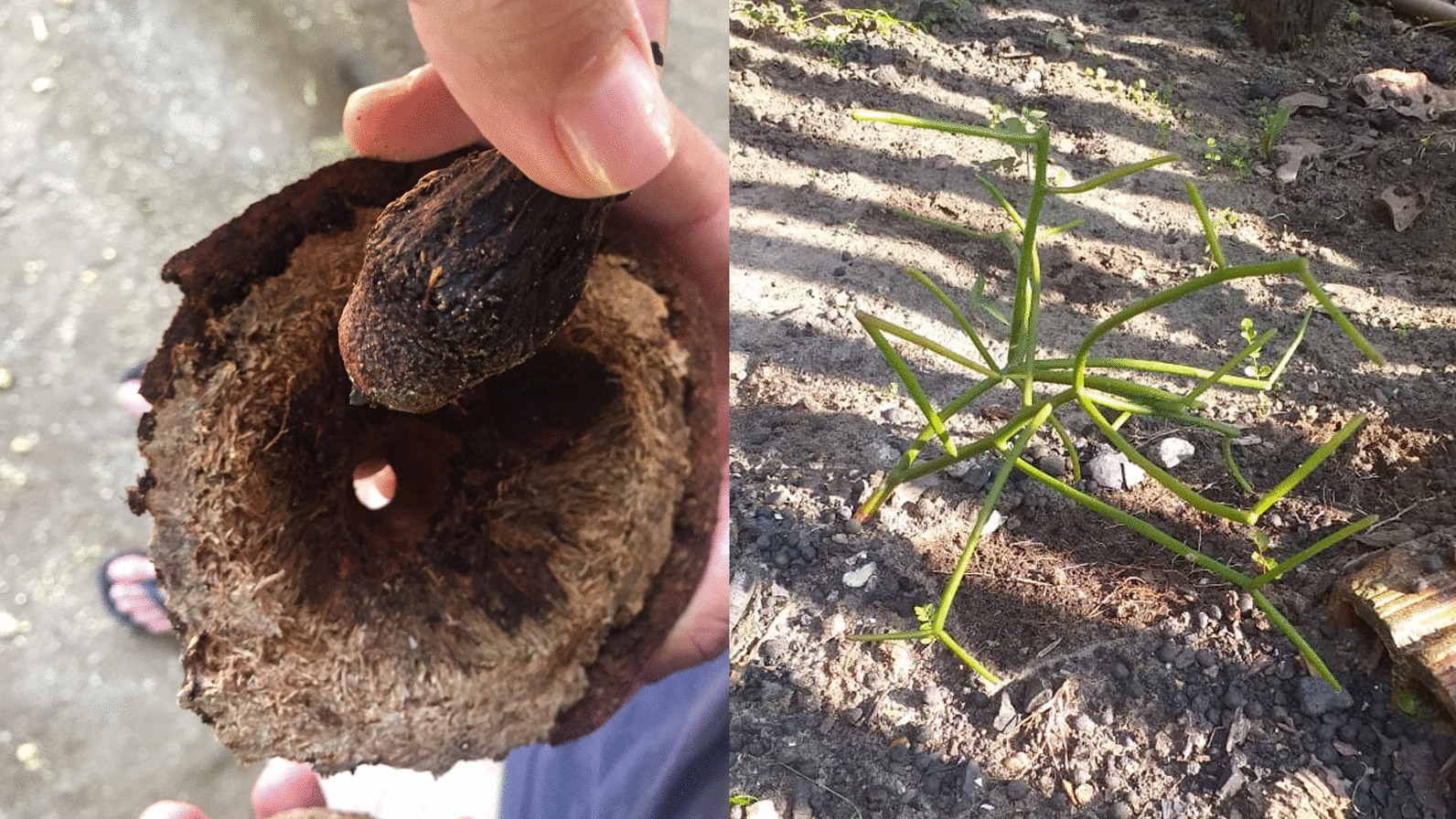
Left: *Umbigo da castanha* (operculum of the Brazil nut; *Bertholletia excelsa* Humb. & Bompl.) cited in Table 1, No. 24 (photograph by the authors, Amanã). Right: Stem architecture of the *cachorro pelado* (pencil cactus; *Euphorbia tirucalli*) cited in No. 55 (photograph: José dos Santos Raimundo Reis, Amanã)

The analogical practices reported here appear to arise from the identification of diverse perceptual and behavioral features of emanator and receiver entities present in the environment that are later applied in specific use settings. This awareness of diverse environmental features, what Tim Ingold [102] refers to as “attention,” [102] encompasses the identification of specific characteristics of plants, animals and abiotic entities as well as a wide range of other practical and intellectual engagements with the environment. We refer to these perceptual characteristics as *activating* features (Fig. 3).

The *emanator* entity has a desired characteristic in relation to the receiver, for example: numerous treatments for dogs that involve administering parts or preparations of predatory animals to make them better hunters (Nos. 1, 6, 8, 11, 16 and 18); the administration of the reproductive organ of the coati as a sexual stimulant (No. 50); or the avoidance of eating watermelon due to its copious red juice by women during menstruation (No. 59).

In some cases, the emanator has some feature related to a third target entity. For example, bats and ants (emanators) that live in groups are administered to a dog (receiver) to improve its ability to corner herd animals (peccaries). In this case, the character of interest can be understood as a way of making the dog attentive to a specific kind of prey. A more direct example of this modality is the practice of blowing the smoke of burnt puma or jaguar fur on a dog to make it a good tracker of large felines (No. 15).

Attributes associated with animal behavior were the most commonly noted in our records. The slow movement of the tortoise and its ability to retract its head are examples of behavioral attributes that were referenced in certain practices (Nos. 25, 27 and 49). *Ribeirinhos* of the Amanã context also perform empirical experiments aimed at determining the suitability of a given specimen for use. For example, hunters in Amanã would butcher a prey animal and wait for insects to arrive to feed on it. The first individual to land on the carcass “has a good sense of smell” and is therefore captured for administration to dogs (analogy 2).

An animal’s *activating* attribute related is not restricted to its natural condition, but may also involve the situation in which it was killed or butchered for consumption. Two examples from Ribeira involved prohibitions about consuming animals captured using a certain kind of trap known as *mundéu*. In this trapping technique, small to medium-sized animals such as armadillo, agouti or paca are attracted to a fenced wooden structure, triggering the fall of a heavy tree trunk that crushes the animal. In the analogies reported, the injury the trap causes to the animal can appear in a person who eats its meat (No. 39) or in an infant whose mother has eaten its meat (No. 38).

Such behavioral activating features also include human behavior toward animals. Five analogies reported in Amanã involve the mistreatment of hunted animals, such that a newborn child suffers as the animal suffered, resembles the abused animal, or is born with the same physical disfigurement to which the animal was subjected (Nos. 28–32). In another case, physical effort exerted by the father causes a child to suffer during gestation (No. 33). This example is part of a widely held set of beliefs, practices and restrictions among Amazonian populations surrounding *couvade*, such that the diet and behavior of father and mother alike can influence the well-being of a gestating and recently born child [103].

The concept of “activators” that we develop here fits in with the broader effort within ethnobiology to understand the function as well as origin of use repertoires. In the field of ethnobotany in particular, the ecological apparency hypothesis [25] has been used to understand medicinal uses of plants [26]. As originally proposed by Feeny [25], plants that are more “apparent” in the environment (larger or more abundant) tend to experience high herbivory rates, thus evolving quantitative (e.g., tannins) or qualitative (e.g., alkaloids) chemical defenses [25]. In an adaptation of apparency theory to ethnobotany, the ecological apparency of plants in conjunction with their pharmacological and biochemical characteristics results in varying degrees of use of different botanical groups by humans [26, 104–106].

Shepard [2] proposes expanding this concept to include “sensory apparency,” namely, the interaction of ecological abundance and biochemical properties with culturally-mediated sensory evaluations of plants including color, texture, odor and taste. In this way, different societies’ theories about illness etiology and plant efficacy, elaborated through different sensory modes, can result in a culturally variable sensory bias, detectable in patterns of use of different plant groups [2, p. 262].

While perceptual cues, especially visual (color, shape, form) are certainly important in the analogical theories described here, salient traits such as intelligence, ferocity, foraging habit and sociality that are identified and activated by *quilombola* and *ribeirinho* “sympathies” go beyond the merely sensorial to encompass more complex aspects of animal behavior and forest ecology. Coupled with analogical reasoning, this attention to habit and behavior, especially in the use of fauna-based remedies, describes a systematic theory of “ethological” apparency in these, and certainly other, ethnozoological repertoires. As is the case with the “Doctrine of Signatures” in ethnobotany [39], such rationalities of usage are typically dismissed as “metaphorical” or “magical” in studies of traditional medicinal uses of fauna [107–110]. In addition to better describing the uses reported in the ethnographic contexts presented here, we suggest that cultural variations on the concept of “apparency” could be applied more broadly to the study of ethnobiological and especially ethnozoological use repertoires.

### Epistemic dimension: grouping entities in analogical chains

The analogical rationality reported here can be understood as an intellectual process that extends from the phenomenology of environmental perception, establishing and elaborating relationships between the entities. In this way, chains or series of otherwise unrelated entities become causally associated by means of the activating feature. In the cited uses, for example, a group of unrelated animals, the *japiim* (yellow-rumped cacique), various primate species and the *tucuxi* dolphin, are all considered to be “very intelligent” animals (Nos. 4, 5 and 8), justifying their use to improve the acuity of hunting dogs in Amanã.

Likewise, bats and army ants both demonstrate social behavior leading to their agglomeration in large groups for roosting and foraging, respectively. Treatments prepared from both types of animal are administered to hunting dogs in Amanã to improve the ability of dogs to track *caititu* (peccaries), which also live and forage in herds (Nos. 9 and 10). A third example, also from Amanã, involves animals recognized as good predators, such as *calangos* (lizards), *vespas* (wasps) and “hunter beetles” (Nos. 1, 6 and 11). These are likewise administered to dogs to improve their hunting abilities.

In the cases exemplified above, organisms as distinctive as birds and aquatic mammals, peccaries and bats, or lizards and wasps are grouped according to perceptually salient, though not taxonomically significant, shared behaviors. These latent, decidedly non-natural groupings appear to contradict prominent theories regarding the culturally universal features of folk biological classification systems [27–29, 32, 33], while reinforcing the critiques of some authors regarding the importance of utilitarian and other culturally variable categories [24, 30, 31, 111].

With regard to cognitive aspects of such classification processes [34], the degree of similarity between entities acts as the basis for an inferential logic behind grouping and classifying different entities into categories. In Western scientific taxonomy, as well as in many works on folk biological taxonomies, the degree of biological affinity between organisms is inferred from the combined criterion of similarity and typicality [33–36]. Yet in the practices reported here, as well as the tacit groupings they evoke, the process of identifying similarities and classifying entities proceeds through a different form of epistemology. Analogical rationality begins with the perception of new similarities between entities, over and above existing categories, that are constantly being produced in a world marked by an emergent “coming into being” of things and organisms that inhabit it. This is possible because, according to analogical reasoning, all characteristics are potentially transferable, thus able to modify the constitution of other entities. This fundamental epistemological feature applies to all of the analogies reported here (Table 1).

Mainstream Western approaches to scientific as well as folk biological taxonomy infer diachronic continuity from a given set of similarities deemed salient for classificatory purposes. This epistemic process, which emerges from a “naturalistic” ontology, is evident for example in concepts like genealogy and evolutionary history in the natural sciences, as well as for arguments about the importance of morphological similarities produced by evolution in studies of folk biology. By contrast, the analogical knowledge practices reported here work in somewhat the opposite fashion, such that similarities are inferred from salient, synchronic continuities (Table 3). This kind of epistemology is inherent in the analogistic ontological framework.

**Table 3:** Contrasting epistemologies: naturalism versus analogism

	Given condition	Inferred condition
Naturalism	Similarity	Diachronic ontological continuity
Analogism	Synchronic ontological continuity	Similarity

### Ontological dimension: a permeable world of interchangeable qualities

The data presented here reveal evidence of an analogistic reasoning process driving ethnobiological use patterns related to subsistence practices and daily life among *quilombola* and *ribeirinho* communities in Brazil (Fig. 2). Analogism acts as a far-reaching epistemic model, or *modus operandi*, in both ethnographic contexts. This form of rationality is the product of an ontological frame in which certain qualities of one entity can be transmitted to another entity, establishing a sympathetic mode of causality such that similarity in quality produces similarity in effect. The condition of transmissibility implies an ongoing flow of properties between different things and beings, including people, animals, plants, meteorological phenomena and material objects. This form of rationality highlights a conception of the world as a place full of permeable beings and things, a world in which the virtues or essences of different entities are interchangeable. It is this conception of reality that Descola [7] categorizes as analogism, in contrast to naturalism, animism and totemism.

In our analysis of the analogical reasoning implied in these ethnobiological practices, we have focused on their potential for identifying and producing similarity between otherwise dissimilar entities. This process depends on a form of causality in which a given entity is susceptible to changing its nature when in contact with the qualities of another entity. We believe that this particular causal aspect is what best defines the ontological difference between these practices and Western naturalistic thought, rather than the varying roles of exteriority vs. interiority as hypothesized by Descola.

In a conception of reality that allows qualities to flow between different entities, their flow can be controlled by administering or avoiding a given entity with some desirable or undesirable quality. In this sense, the analogy is first identified or projected, and then the effect of contact between the entities is produced by facilitating or preventing the transfer of a quality from one entity to another. In a world (or cultural reality) where distance does not prevent transmission [50], the uses, practices and prohibitions described here represent a logical consequence of living, thinking and acting in that world.

### Toward a historical understanding of analogism in Brazilian ethnobiological repertories

We were particularly struck by the tremendous similarities in the analogical rationality at work in these geographically distant, ecologically distinctive and demographically unrelated populations, including certain nearly identical use patterns, for example, the administration of wasp preparations to hunting dogs. It is of course possible that certain general concepts or specific ethnobiological practices were absorbed through syncretism with indigenous populations that occupied these regions prior to colonization. However, the geographical distance would preclude a common origin of any specific cultural knowledge. Moreover, and in contrast to the *ribeirinho* and *quilombola* cases presented here, indigenous Amazonian use rationales that follow an apparently analogical or homeopathic pattern of “like treats like” reveal a decidedly animistic logic of sympathetic transmission mediated by spiritual “owners” or “masters” [2, 4].

It is also possible that similar concepts and practices might emerge independently, despite the ecological and geographical distance. However, we suggest that the observed similarities in these ethnobiological repertoires reflects the pervasive historical presence of colonial Portuguese cultural influences in the genesis of these otherwise distinctive social formations. Analogical or “sympathetic” reasoning, which originated in ancient Greece, was dominant throughout Europe until at least the eighteenth century, and was especially prevalent on the Iberian Peninsula, where Greek influences were expressed doubly through European as well as Arabic sources, notably in the context of religious and medical practices [7, 33, 50, 112, 113].

The widely documented classification of “hot” vs. “cold” foods, illnesses and remedies in Brazilian folklore, as well as the concept of *reima* in the Brazilian Amazon, is clearly a reflection of Hippocratic-Galenic humoral theory [113–117] as transmitted through Portuguese colonial influences [3, 103, 113, 118, 119]. These theories were brought to South America during the sixteenth century through Iberian colonization of the New World, and dominated popular medicine in Brazil until the early twentieth century [113]. While humoral theory is widespread in traditional and indigenous medical systems of Meso-America and the Andes [7, 114, 115], “hot–cold” illness concepts are notably absent in the traditional medical systems of some Amazonian indigenous peoples [2], probably reflecting varying degrees of contact and syncretism with Iberian colonial influences.

If we assume that analogism among *quilombolas* in southeast Brazil and *ribeirinhos* in Amazonia is primarily a legacy of Portuguese colonization, we would expect to find an “analogical” signature in the ethnobiological repertoires of rural populations throughout Brazil. A cursory review of ethnobiological studies among rural Brazilian communities, especially zootherapeutic practices, indeed reveal a suggestive prevalence of analogical uses [108–110, 120]. This suggests avenues for future comparative research within Brazil and more widely throughout the Iberian sphere. More generally, the inclusion of explicitly comparative, historical methods might contribute to ethnobiology’s growing interface with historical approaches in ethnobiology [121, 122].

## Conclusion

In this article, we have analyzed the analogical rationalities that govern ethnobiological use repertories among *quilombola* communities of the Atlantic forest and *ribeirinhos* of the Brazilian Amazon. Going beyond a mere listing of species and their uses, we have explored the phenomenological, epistemic, and ontological components that shape peoples’ environmental experiences and interactions. The analogistic practices we report seem to manipulate the flow of qualities among different things and beings in the world by recognizing that certain salient features of these entities are external signs of potentially transmissible qualities. We understand the *simpatias* used by *quilombolas* and *ribeirinhos* alike as a way of exerting control over important phenomena, beings and things in their environment, a systematic *technology*, as opposed to a haphazard collection of superstitions, that is fine-tuned to their ecological surroundings and ontological assumptions.

We propose that the origin of analogical reasoning in these two geographically distant rural communities is due in part to the history of Portuguese colonization. In this way, we link the historical dimension of the study to its phenomenological, epistemological, and ontological components. We believe that our study provides a model for expanding the scope of ethnobiological research among rural populations of Brazil, which to date has been largely concerned with species diversity and knowledge preservation on a case-by-case basis, with little attention to epistemic and ontological dimensions, historical processes or comparative methods.

The analytical method we develop here might contribute more broadly to emerging approaches in multispecies ethnography [57] and “anthropology beyond the human” [42]. Many authors have theorized how nonhuman species can be seen as agents that cohabit multispecies landscapes [58, 59], or even partake of an expanded animistic or “perspectivist” notion of humanity [14, 123]. We contribute to these debates by providing methodological and theoretical tools for analyzing how different species can be understood to take part in semiotic networks of shared characters, properties and powers, thus shaping a “world in the making” [60, 61]. Our methods and conceptual framework might also contribute to more systematic comparative studies in ethnobiology, ecosemiotics [42, 124] and environmental perception [125]. Finally, we hope our study might contribute to greater appreciation of traditional knowledge systems and practices guided by epistemologies and ontologies different from those of hegemonic Western science [7].

## Data Availability

Not applicable.
